# Supplementary material for: Mapping of the gene network that regulates glycan clock of ageing
Source: Aging (Albany NY). 2023 Dec 26;15(24):14509–52. doi: 10.18632/aging.205106 (PMC10781487; doi:10.18632/aging.205106)
Supplement: Supplementary Methods [file aging-15-205106-s001.pdf]

SUPPLEMENTARY METHODS

Studied cohorts

Recruitment of participants, sample collection, genotyping and phenotyping in the cohorts used in the galactosylation GWAS was performed by staff members at the University of Zagreb, Croatia, University of Split Medical School, Croatia, UK, United Kingdom, King's College London, UK, German Institute of Human Nutrition, Germany, University of Tartu, Estonia, Leiden University Medical Centre (LUMC), Netherlands, Helmholtz Zentrum München – German Research Center for Environmental Health, Germany, and the nine study centers of the German Chronic Kidney Disease (GCKD) study.

IgG N-glycan quantification was performed by Genos Glycoscience Research Laboratory, Zagreb, Croatia and LUMC, Leiden, Netherlands.

TwinsUK

TwinsUK is a national registry of 12,000 volunteer twins in the UK. The cohort consists of 83% female subjects with a nearly equal number of monozygotic (51%) and dizygotic (49%) twin pairs. With an aim to study the genetics of healthy ageing and complex diseases, a sample of 7000 twins was assessed for a range of clinical, biochemical, behavioural and socio-economic characteristics. Moreover, several omics' datasets for the TwinsUK dataset are available including genome-wide SNP data. The study participants provided informed consent and ethical approval was obtained for academic and commercial use of the study [1].

The European Prospective Investigation into Cancer and Nutrition

The European Prospective Investigation into Cancer and Nutrition (EPIC) -Potsdam is a prospective cohort study that includes 27548 participants recruited from the Potsdam population in Germany from 1994 to 1998 [2]. Participants' age at recruitment ranges between 35 and 65, and the number of male and female subjects is 10904 and 16644, respectively. Initial data collection consisted of anthropometric measurements and blood sample collection used for omics' data derivation [3]. Ethical approval was obtained from the ethics committee in Germany and all participants provided informed consent [2].

CROATIA Vis, CROATIA-Split and CROATIA-Korcula

CROATIA-Vis, CROATIA-Korcula and CROATIA-Split cohorts were collected as part of the "10001 Dalmatians" study, a study of Croatian island isolates with participants from six Adriatic islands (Korčula, Vis, Lastovo, Susak, Rab, Mljet) and the city of Split. The aim of the study is to investigate genetic and environmental determinants in health and disease by using the advantage of genetically isolated populations. In the recruitment process, a total of 1008 participants aged 19-93 were recruited for the CROATIA-Vis cohort in villages of Vis and Komiza during 2003 and 2004, 1012 subjects aged 18-85 were recruited in 2009-2010 in the city of Split for CROATIA-Split cohort and data on CROATIA-Korcula subjects (aged 18-98) was collected from the island of Korcula, specifically from the town of Korcula and three villages including Lumbarda, Zrnovo and Racisce. Participants were assessed for a number of anthropometric and physiological measurements, and they donated overnight fasting blood samples which were later used for DNA analysis, biochemical measurements and molecular marker assessment [4]. The study participants provided signed informed consent. Ethical approval was obtained for each cohort from ethics committees in Croatia and Scotland.

The Orkney Complex Disease Study

The Orkney Complex Disease Study (ORCADES) is a family-based cohort collected with the goal of identifying genetic risk factors in complex diseases in the population of isolated Orkney Island in northern Scotland. The recruitment started in 2005 and lasted for six years during which 2080 participants were recruited. The subjects were included if they had at least two of their grandparents who were Orcadian. The initial data collection included cardiovascular measurements and fasting blood sample collection, followed by subsequent visits for cognitive function assessment, eye measurements and DEXA scans. The study was approved by ethics committees in Scotland and all participants gave informed consent [5].

Leiden Longevity Study

Leiden Longevity Study (LLS) is a family-based cohort from the Dutch population which was intended for studies of human longevity. Nonagenarian siblings (individuals having a sibling older than 89 years for men and 91 years for women) and their offspring and offspring's spouses (serve as controls) were included in the study if they were European. Initial recruitment

started in 2002 and ended in 2006 during which blood samples were collected for assessment of plasma parameters and genetic material extraction. A total of 3359 subjects were included: 944 long-lived proband siblings, 1671 offspring and 744 controls (offspring's spouses). This study was approved by the ethics committee of LUMC, Netherlands. Signed informed consent was given by all participants [6]. In the current study, a subset of 1190 participants including only offspring and their spouses is used.

The Cooperative Health Research in the Augsburg Region F4

The Cooperative Health Research in The Augsburg Region (KORA) F4 is a population-based study that was conducted between 2006 and 2008 as a follow-up of the KORA S4 study [7]. Participants were randomly selected from the population registry in the Augsburg region and two other neighbouring counties in Germany. The data collection included standard medical and physical examinations. A total of 3080 participants (51% females) aged 32-86 years were recruited [8]. Ethical approval for the study was obtained from the Ethics committee of Bavarian Chamber of Physicians, Germany. All participants provided signed informed consent before entering the study.

The Viking Health Study - Shetland

The Viking Health Study - Shetland (VIKING) is an epidemiologic study initiated to explore genetic risk factors for complex diseases. The cohort consists of individuals from an isolated population of Shetland in northern Scotland and the main criteria for recruitment was to have at least two grandparents from Shetland. Between 2013 and 2015, 2105 participants were recruited. A large number of distant relatives makes the VIKING cohort fit for the identification of rare genetic variants influencing the disease risk [9]. During initial data collection, data on health-related phenotypes and environmental parameters were collected and participants donated a fasting blood sample.

The Estonian Genome Center of the University of Tartu

The Estonian Genome Center of the University of Tartu (EGCUT) Biobank is a volunteer-based cohort of 52 thousand adult subjects aged ≥ 18 from the Estonian population. The recruitment was conducted throughout Estonia via general practitioners' offices and medical personnel during the 2002-2012 period. Besides completing a questionnaire on topics such as lifestyle, diet and clinical diagnostics, participants also donated blood samples. The cohort was utilized in the exploration of more than 200 traits including anthropometric traits, common and rare diseases, blood biochemistry, as well as lifestyle and personality traits. The data on study participants is being continuously updated via follow-up health checks using electronic health registries and re-examinations [10].

German Chronic Kidney Disease study

German Chronic Kidney Disease (GCKD) study is an ongoing prospective study of kidney disease patients who are under nephrologist care in Germany [11]. The current sample size of 5217 makes it one of the largest chronic kidney disease cohort in the world. The subject enrolment was undertaken between 2010 and 2012 via nephrologist practice and outpatient care units of nine study centers throughout different regions in Germany. The mean age of study subjects is 60 years, with 40% of the participants being female. The data collection includes collecting information on sociodemographic factors, medical and family history, as well as obtaining blood samples in a standardized manner, which we immediately processed and stored frozen in a central biobank until measurement of the glycans.

Genotyping, genotype QC and genotype imputation

Genotyping was performed using commercially available SNP genotyping arrays, followed by genotype calling in Illumina and Genome Browser software**.** Quality control (QC) of genotype data was performed to exclude SNPs and samples with low quality including removal of 1) SNPs with low call rate, 2) SNPs violating the assumptions of Hardy-Weinberg Equilibrium (HWE) and 3) SNPs with low minor allele frequency (MAF) < 1%. Details on SNP exclusion criteria for each cohort and imputation to Haplotype Reference Consortium (HRC) [12] panel are shown in Appendix Table 8. The genotypes were mapped to Genome Reference Consortium GRCh37 (hg19) build.

IgG N-glycome analysis

IgG N-glycan quantification by ultra-performance liquid chromatography

Ultra-performance liquid chromatography (UPLC) is used for quantification of glycan structures attached to Fc (constant region) and Fab (variable region) portions of IgG without the possibility to distinguish them. Detailed protocol for UPLC quantification of IgG N-glycans is published elsewhere [13].

Briefly, IgG was isolated from blood plasma samples using Protein G plates (BIA Separations, Ajdovščina, Slovenia). After filtration, plates were extensively washed to remove unwanted proteins and IgG was

released from protein G monoliths using 0.1 M formic acid. Eluates were collected in a 96-well plate and neutralized with neutralization buffer (1 M ammonium bicarbonate) to pH 7.0 to maintain the stability of IgG. IgG samples were dried and denatured using SDS detergent and incubated at 65° C for 10 minutes. N-glycans from IgG were released using recombinant N-glycosidase F followed by fluorescent labelling with 2-aminobenzamide (2-AB) dye. Hydrophilic interaction liquid chromatography (HILIC) based solid-phase extraction (SPE) was used to remove excess protein, reagents and fluorescent label. Fluorescently labelled N-glycans were separated by hydrophilic interaction UPLC on Waters Aquity UPLC H-class instrument (Waters, Milford, MA) with Waters bridged ethylene hybrid (BEH) glycan chromatography column. A linear gradient of 75 to 62% ACN in a 20-min analytical run was used to separate different glycan structures. The retention times for individual glycans were converted to glucose units based on hydrolysed and 2-AB labelled glucose oligomers which were used as external standards for calibration of the system. Data processing was done in two ways depending on the cohort, 1) automatic integration as described in Agakova et al. [14] or 2) using Empower 3 software with an automated processing method with traditional integration algorithm, followed by manual correction of each chromatogram to maintain the same integration intervals in all samples. The resulting chromatograms were separated into 24 peaks where the amount of glycans was expressed as % of the total integrated area in the corresponding peak (GP1-GP24). Total separation of each glycan structure is not possible using the described method, thus resulting in multiple glycan structures being detected under ten peaks. Glycan structures in each peak are listed in Appendix Table 9.

Glycan quantification by liquid chromatography coupled with mass spectrometry

The full name of the method is reverse-phase nano-liquid-chromatography-sheath-flow-electrospray-mass spectrometry (LC-ESI-MS) but in this study, we refer to it as LC-MS. The detailed protocol for analysis of IgG N-glycans using LC-MS is described in Selman et al. [15]. Briefly, IgG was isolated by affinity chromatography binding to protein G 96-well plates (BIA Separation, Ajdovščina, Slovenia) and treated with trypsin overnight at 37° C which allowed cleavage of IgG at specific amino acid sites. The cleavage by trypsin resulted in different glycopeptides due to the difference of amino acid sequence in different IgG subclasses, thereby enabling subclass-specific glycan measurements. IgG subclass separation was performed using the Ultimate 3000 HPLC system (Dionex Corporation, Sunnyvale, CA). The SPE trap column was conditioned with mobile phase A and samples were loaded and separated on Ascentis Express C18 nano-LC column (Supelco, Bellefonte, USA) conditioned with mobile phase A and 95% ACN. For detection of separated subclass-specific glycopeptides, the HPLC system was coupled to a Dionex Ultimate UV detector and interfaced to a quadrupole-TOF-MS mass spectrometer (Bruker Daltonics, Bremen, Germany) with a standard ESI source (Bruker Daltonics, Bremen, Germany) and a sheath-flow ESI sprayer (Agilent Technologies, Santa Clara, USA). The mass spectra were recorded in a range between 300 and 2000 m/z with two averages at the frequency of 1Hz. The analysis time for one sample was 16 minutes. The calibration of LCMS datasets was done internally using a list of known glycopeptides and datasets were exported to the open mzXML format by Bruker DataAnalysis 4.0 software, followed by alignment to a master dataset of a typical sample. In-house software "Xtractor2D" was used to extract pre-defined features such as peak maximum or peak area in specific retention time and mass windows. Relative intensities of subclass-specific glycopeptides were obtained by integrating and summing three isotopic peaks. The obtained intensities were then normalized to the total IgG subclass-specific glycopeptide intensities. IgG2 and IgG3 subclasses have the same tryptic glycopeptide moieties, thus not enabling the separation of the subclass-specific glycopeptides. Here, obtained measurements are simply referred to as IgG2/3. LC-MS quantification results in 50 values which refer to 20 glycans measured on IgG1, 20 glycans on IgG2/3 and 10 glycans on IgG4. All glycans measured on IgG4 are fucosylated structures since the nonfucosylated glycans are hard to distinguish from the glycans found on IgG1 [16]. The list of glycans measured by LC-MS and their description is listed in Appendix Table 10.

IgG glycan data harmonization

Previously, there were no GWA meta-analyses of IgG N-glycan patterns using GWAS of both UPLC- and LC-MS-derived IgG N-glycan traits, therefore making it necessary to first assess the correlation of the data and methods which should be applied in pre-processing step to make UPLC and LC-MS glycan traits comparable. For this purpose, we used the CROATIA-Vis cohort (n=661) as both UPLC and LC-MS IgG N-glycan measurements are available in the same samples.

We aimed to combine IgG subclass information obtained from LC-MS in an appropriate manner to obtain information corresponding to total IgG glycome values measured by UPLC. Pre-processing of IgG glycome data consists of normalization of the data and batch correction to remove the effects of experimental variation. Normalization procedure is necessary to remove

unwanted variation between the samples and allows quantitative comparison of the samples [17]. We tested the following three normalization types: total area normalization, largest peak normalization and median quotient normalization, all of which can be applied using “glycanr” [18] package in R software [19]. We tested different normalizations both across the total glycome and per IgG subclass in LC-MS data.

Due to varying laboratory conditions during IgG N-glycan measurement, it was necessary to perform batch correction to remove non-biological, experimental variation. The batch correction was performed using ComBat function in R package “sva” [20]. We first log-transform the data as ComBat function implements empirical Bayes method for batch correction [21] which assumes a normal distribution of the data, followed by batch correction with ComBat() where the batch is denoted by the plate on which the samples were analysed, and lastly, exponential transformation of the values to the original scale.

We calculated derived traits from the initial traits for purpose of data harmonization and to enable a more straightforward interpretation of the GWAS results so that the discovered genomic loci can be directly linked to the addition of one or two galactose residues to the IgG N-glycan chain: agalactosylation (G0), monogalactosylation (G1) and digalactosylation (G2). Formulas for calculation are listed in Appendix Table 11.

Since LC-MS data quantifies glycans attached to different IgG subclasses, in order to calculate different galactosylation traits we also incorporated the approximation of the IgG glycan subclass response factor (RF) to represent the IgG subclass concentration relative to other subclasses. RF is defined as the ratio between the concentration of the analyte and instrument response to the analyte. Using unpublished in-house experimental data, the subclass-specific response factors were approximated as follows: IgG1 with RF=1, IgG2/IgG3 with RF=2 and IgG4 with RF=1. IgG subclasses are present in different quantities in human serum; hence we also incorporated relative concentrations of each IgG subclass in the calculation of derived traits. The following relative measurements were indicated in the literature: 66% for IgG1, 30% for IgG2/IgG3 and 4% for IgG4 [22]. The subclass-specific glycan measurements were weighted by the corresponding concentration and response factor before trait calculation.

Pre-processing of glycan data

Glycan data was pre-processed centrally in Genos for all cohorts except the LLS cohort for which the glycan data was pre-processed by a colleague from Leiden University Medical Centre. It is important to note that glycan data for the CROATIA-Korcula cohort was obtained in three instances (2010, 2013 and 2017) and each dataset was pre-processed separately and treated as an individual cohort in downstream analysis. Additionally, the TwinsUK cohort was analysed in four separate batches. Due to differences in sample collection, batches 1 and 2 were treated as one dataset and batches 3 and 4 were treated as the second dataset.

Data points in the 99.9th percentile were removed and considered as technical outliers. Next, based on the results of the previous data harmonization assessment, median quotient normalization was applied on both UPLC and LC-MS glycan data across 24 and 50 glycan measurements, respectively and the batch correction was applied. Prior to the genetic association test, galactosylation traits in all cohorts were transformed using rank-based inverse normal transformation (mean=0, standard deviation=1).

SUPPLEMENTARY REFERENCES

1. Moayyeri A, Hammond CJ, Hart DJ, Spector TD. The UK Adult Twin Registry (TwinsUK Resource). Twin Res Hum Genet. 2013; 16:144–9. https://doi.org/10.1017/thg.2012.89 PMID:23088889
2. Boeing H, Korfmann A, Bergmann MM. Recruitment procedures of EPIC-Germany. European Investigation into Cancer and Nutrition. Ann Nutr Metab. 1999; 43:205–15. https://doi.org/10.1159/000012787 PMID:10592369
3. Bergmann MM, Bussas U, Boeing H. Follow-up procedures in EPIC-Germany--data quality aspects. European Prospective Investigation into Cancer and Nutrition. Ann Nutr Metab. 1999; 43:225–34. https://doi.org/10.1159/000012789 PMID:10592371
4. Campbell H, Carothers AD, Rudan I, Hayward C, Biloglav Z, Barac L, Pericic M, Janicijevic B, Smolej-Narancic N, Polasek O, Kolcic I, Weber JL, Hastie ND, et al. Effects of genome-wide heterozygosity on a range of biomedically relevant human quantitative traits. Hum Mol Genet. 2007; 16:233–41. https://doi.org/10.1093/hmg/ddl473 PMID:17220173
5. McQuillan R, Leutenegger AL, Abdel-Rahman R, Franklin CS, Pericic M, Barac-Lauc L, Smolej-Narancic N, Janicijevic B, Polasek O, Tenesa A, Macleod AK, Farrington SM, Rudan P, et al. Runs of homozygosity in European populations. Am J Hum Genet. 2008; 83:359–72. https://doi.org/10.1016/j.ajhg.2008.08.007 PMID:18760389
6. Schoenmaker M, de Craen AJ, de Meijer PHE, Beekman M, Blauw GJ, Slagboom PE, Westendorp RGJ. Evidence

of genetic enrichment for exceptional survival using a family approach: the Leiden Longevity Study. Eur J Hum Genet. 2006; 14:79–84. https://doi.org/10.1038/sj.ejhg.5201508 PMID:16251894

7. Holle R, Happich M, Löwel H, Wichmann HE, and MONICA/KORA Study Group. KORA--a research platform for population based health research. Gesundheitswesen. 2005 (Suppl 1); 67:S19–25. https://doi.org/10.1055/s-2005-858235 PMID:16032513

8. Rathmann W, Kowall B, Tamayo T, Giani G, Holle R, Thorand B, Heier M, Huth C, Meisinger C. Hemoglobin A1c and glucose criteria identify different subjects as having type 2 diabetes in middle-aged and older populations: the KORA S4/F4 Study. Ann Med. 2012; 44:170–7. https://doi.org/10.3109/07853890.2010.531759 PMID:21091229

9. Halachev M, Meynert A, Taylor MS, Vitart V, Kerr SM, Klaric L, Consortium SGP, Aitman TJ, Haley CS, Prendergast JG, Pugh C, Hume DA, Harris SE, Liewald DC, et al. Increased ultra-rare variant load in an isolated Scottish population impacts exonic and regulatory regions. PLoS Genet. 2019; 15:e1008480. https://doi.org/10.1371/journal.pgen.1008480 PMID:31765389

10. Leitsalu L, Haller T, Esko T, Tammesoo ML, Alavere H, Snieder H, Perola M, Ng PC, Mägi R, Milani L, Fischer K, Metspalu A. Cohort Profile: Estonian Biobank of the Estonian Genome Center, University of Tartu. Int J Epidemiol. 2015; 44:1137–47. https://doi.org/10.1093/ije/dyt268 PMID:24518929

11. Titze S, Schmid M, Köttgen A, Busch M, Floege J, Wanner C, Kronenberg F, Eckardt KU, and GCKD study investigators. Disease burden and risk profile in referred patients with moderate chronic kidney disease: composition of the German Chronic Kidney Disease (GCKD) cohort. Nephrol Dial Transplant. 2015; 30:441–51. https://doi.org/10.1093/ndt/gfu294 PMID:25271006

12. Loh PR, Danecek P, Palamara PF, Fuchsberger C, A Reshef Y, K Finucane H, Schoenherr S, Forer L, McCarthy S, Abecasis GR, Durbin R, L Price A. Reference-based phasing using the Haplotype Reference Consortium panel. Nat Genet. 2016; 48:1443–8. https://doi.org/10.1038/ng.3679 PMID:27694958

13. Pucić M, Knezević A, Vidic J, Adamczyk B, Novokmet M, Polasek O, Gornik O, Supraha-Goreta S, Wormald MR, Redzić I, Campbell H, Wright A, Hastie ND, et al. High throughput isolation and glycosylation analysis of IgG-variability and heritability of the IgG glycome in three isolated human populations. Mol Cell Proteomics. 2011; 10:M111.010090. https://doi.org/10.1074/mcp.M111.010090 PMID:21653738

14. Agakova A, Vučković F, Klarić L, Lauc G, Agakov F. Automated Integration of a UPLC Glycomic Profile. Methods Mol Biol. 2017; 1503:217–33. https://doi.org/10.1007/978-1-4939-6493-2_17 PMID:27743370

15. Selman MHJ, Derks RJE, Bondt A, Palmblad M, Schoenmaker B, Koeleman CAM, van de Geijn FE, Dolhain RJE, Deelder AM, Wuhrer M. Fc specific IgG glycosylation profiling by robust nano-reverse phase HPLC-MS using a sheath-flow ESI sprayer interface. J Proteomics. 2012; 75:1318–29. https://doi.org/10.1016/j.jprot.2011.11.003 PMID:22120122

16. Huffman JE, Pučić-Baković M, Klarić L, Hennig R, Selman MHJ, Vučković F, Novokmet M, Krištić J, Borowiak M, Muth T, Polašek O, Razdorov G, Gornik O, et al. Comparative performance of four methods for high-throughput glycosylation analysis of immunoglobulin G in genetic and epidemiological research. Mol Cell Proteomics. 2014; 13:1598–610. https://doi.org/10.1074/mcp.M113.037465 PMID:24719452

17. Karaman I. Preprocessing and Pretreatment of Metabolomics Data for Statistical Analysis. Adv Exp Med Biol. 2017; 965:145–61. https://doi.org/10.1007/978-3-319-47656-8_6 PMID:28132179

18. Ugrina I, Klaric L, Vuckovic F, Russell A. glycanr: Tools for Analysing N-Glycan Data [Internet]. 2018.

19. R Core Team. R: A language and environment for statistical computing. [Internet]. Vienna, Austria: R Foundation for Statistical Computing. 2017.

20. Leek JT, Johnson WE, Parker HS, Jaffe AE, Storey JD. The sva package for removing batch effects and other unwanted variation in high-throughput experiments. Bioinformatics. 2012; 28:882–3. https://doi.org/10.1093/bioinformatics/bts034 PMID:22257669

21. Johnson WE, Li C, Rabinovic A. Adjusting batch effects in microarray expression data using empirical Bayes methods. Biostatistics. 2007; 8:118–27. https://doi.org/10.1093/biostatistics/kxj037 PMID:16632515

22. Vidarsson G, Dekkers G, Rispens T. IgG subclasses and allotypes: from structure to effector functions. Front Immunol. 2014; 5:520. https://doi.org/10.3389/fimmu.2014.00520 PMID:25368619
