# Supplementary material for: Mapping of the gene network that regulates glycan clock of ageing
Source: Aging (Albany NY). 2023 Dec 26;15(24):14509–52. doi: 10.18632/aging.205106 (PMC10781487; doi:10.18632/aging.205106)
Supplement: Supplementary Figures [file aging-15-205106-s002.pdf]

SUPPLEMENTARY FIGURES

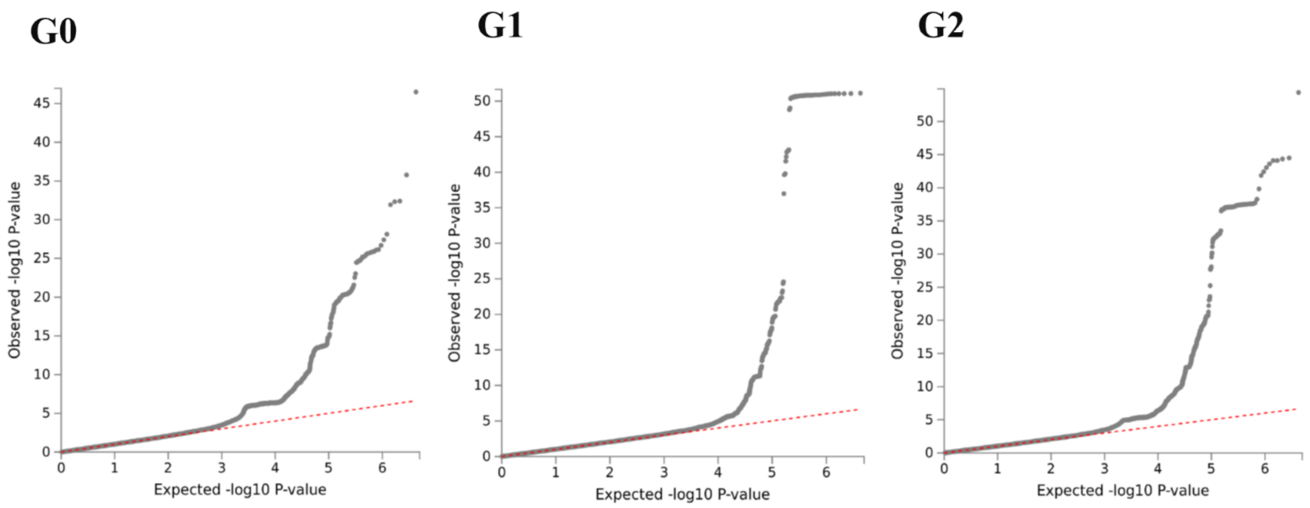

Supplementary Figure 1. QQ plots of association analysis for G0, G1 and G2 traits. Genomic inflation (lambda) for meta-analysis was 1.02, 1.01 and 1.03 for G0, G1 and G2, respectively.

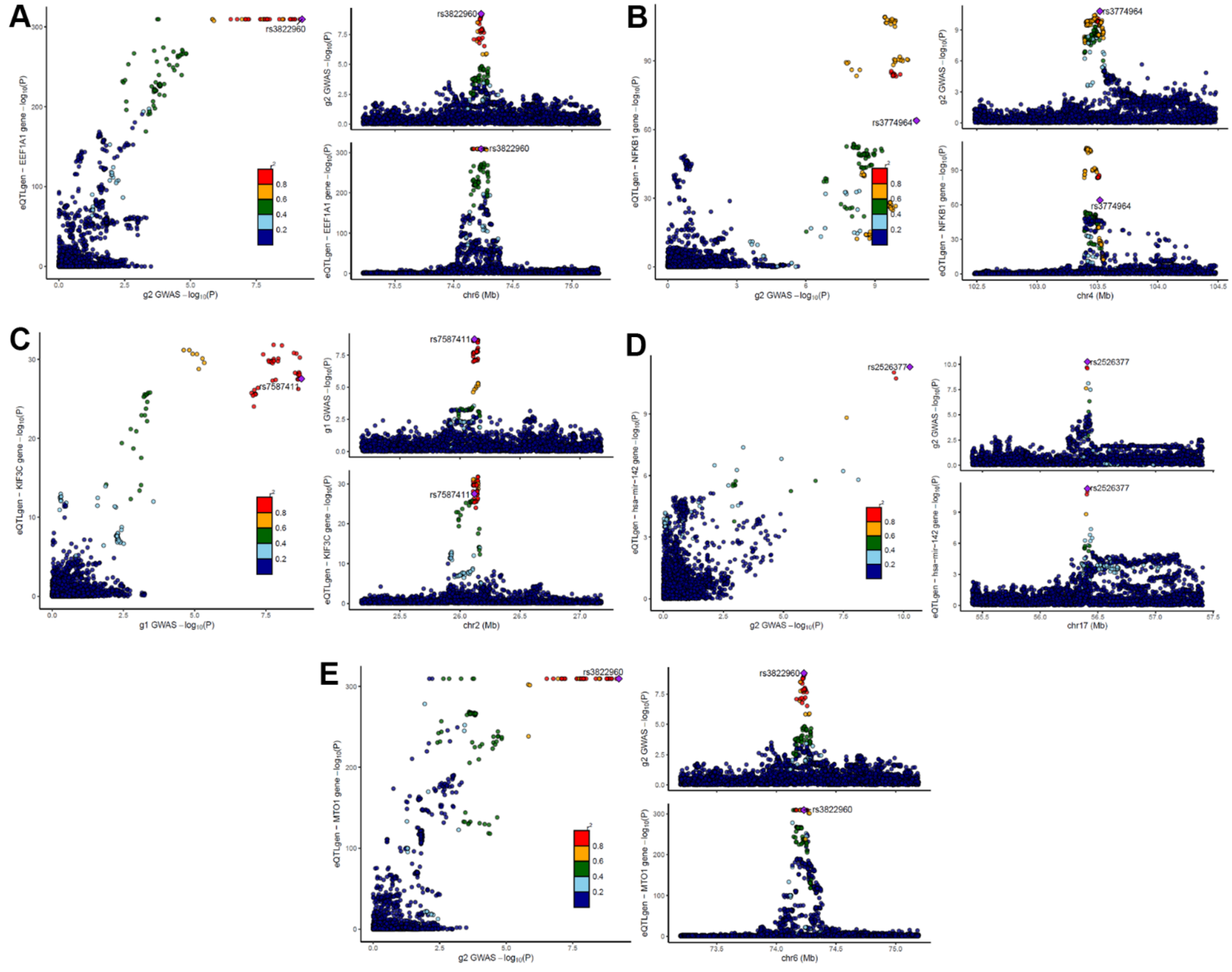

Supplementary Figure 2. IgG galactosylation and eQTL association plots at loci with colocalization (PP4 > 75%). Association plot for IgG galactosylation GWAS and eQTL are shown in the top right and bottom right corners, respectively; x-axis shows the chromosomal position, y-axis shows -log10(p-value) of the association. Correlation of -log10(p-values) for IgG galactosylation GWAS and eQTL are shown on left side. (**A**) Colocalization in *EEF1A1* locus (prioritized genes *EEF1A1* and *MTO1*), (**B**) Colocalization in *NFKB1* locus (prioritized genes *NFKB1* and *MANBA*), (**C**) Colocalization in *KIF3C* locus (prioritized gene *KIF3C*), (**D**) Colocalization in *hsa-mir-142* locus (prioritized genes *BZRAP1*, *SUPT4H1* and *RAD5C1*), (**E**) Colocalization in *MTO1* locus (prioritized genes *EEF1A1* and *MTO1*).

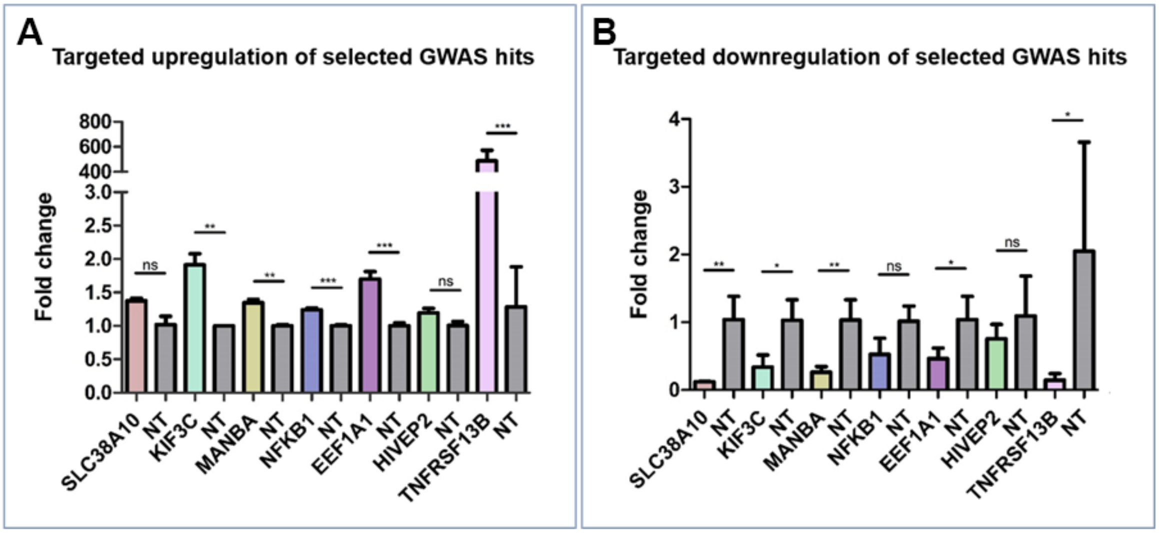

Supplementary Figure 3. Upregulation and downregulation of selected GWAS hits. Selected GWAS hits *associated* with IgG galactosylation (*SLC38A10, KIF3C, MANBA, NFKB1, EEF1A1, HIVEP2* and *TNFRSF13B*) were targeted in dCas9-VPR and dCas9-KRAB monoclonal cell lines. Samples containing non-targeting gRNAs served as controls. Changes of transcript levels are given as fold change values. (**A**) Targeting of selected GWAS hits with specific gRNA molecules in dCas9-VPR cell line resulted in significant increase of transcript levels of all loci except for *SLC38A10* and *HIVEP2*. (**B**) Targeting of the same genes in dCas9-KRAB call line resulted in significant downregulation of all genes except for *NFKB1* and *HIVEP2.* Statistical significance: *<0.05; **<0.01; ***<0.001; ns, not significant.

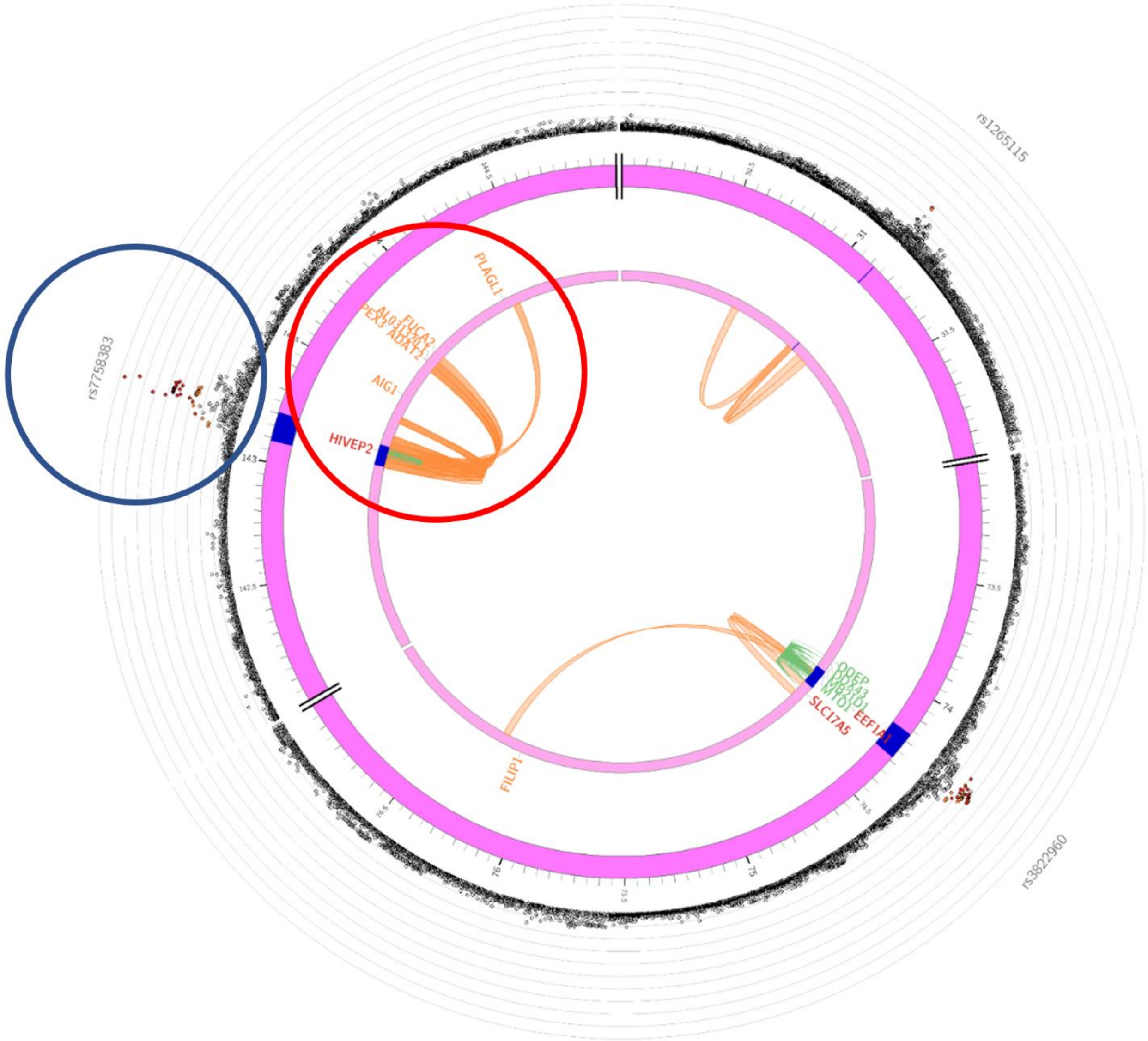

Supplementary Figure 4. Chromatin interaction map based on Hi-C data for GM12878 cell line (GSE87112) for chr6:143088071-143206826 locus indicating *FUCA2* and 4 other genes mapped via chromatin interaction with genetic variants in *HIVEP2* locus. Blue circle denotes the defined genomic locus; red circle denotes genes mapped via chromatin interaction mapping by FUMA.

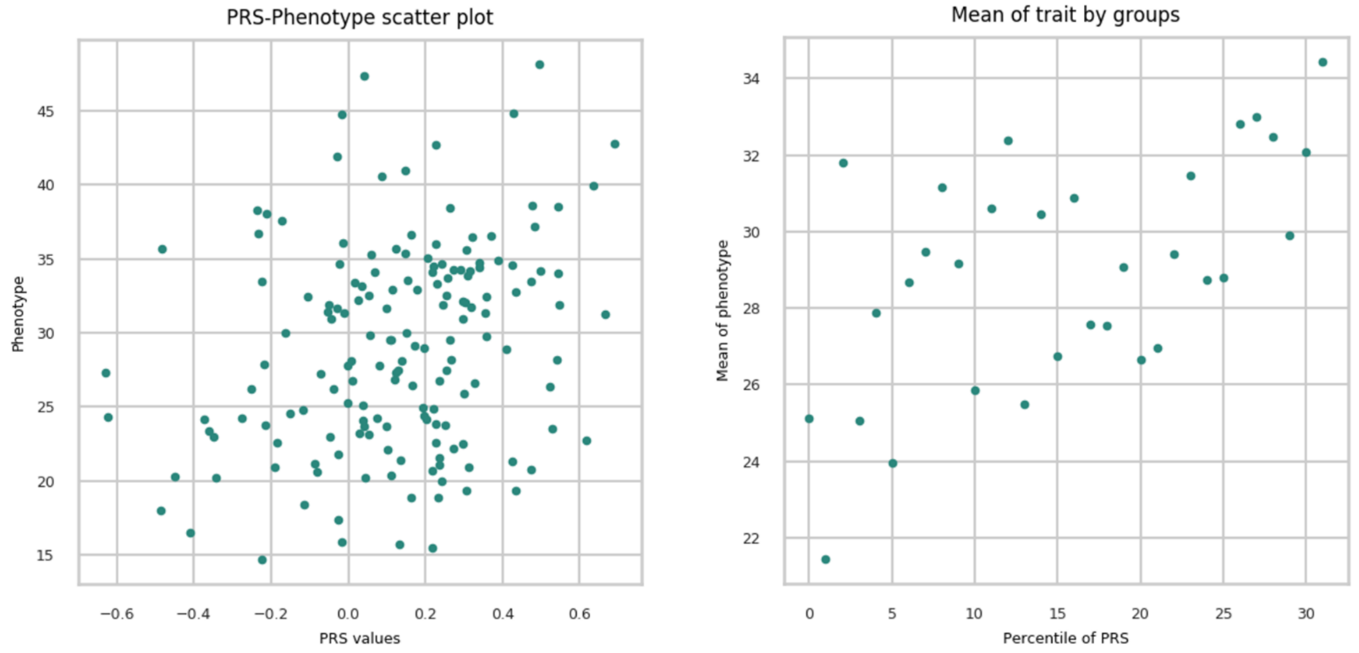

Supplementary Figure 5. PRS-Phenotype scatter plot (G0) and mean of trait by groups. The graph on the left-hand side shows the dependence of phenotype values (G0) on PRS values. The graph on the right-hand side is data stratified into 30 groups according to the PRS percentile. The Y-axis shows the average value of the trait for the group, the X-axis shows the group number.

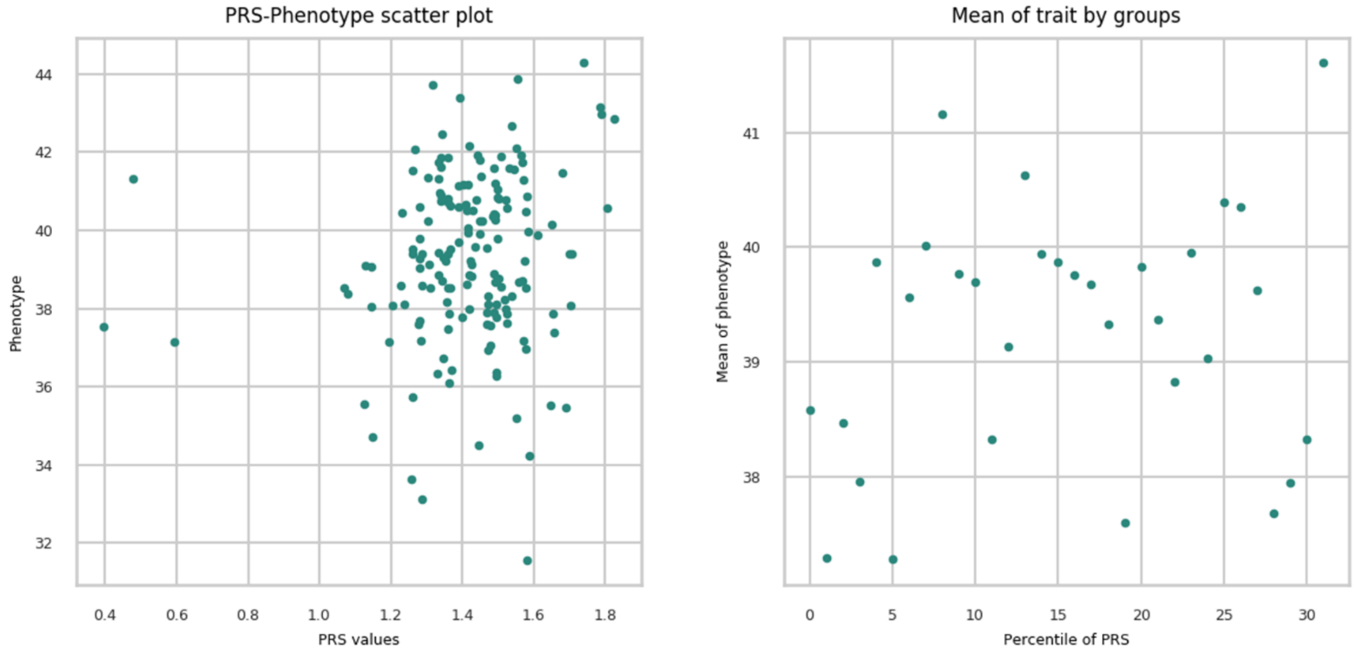

Supplementary Figure 6. PRS-Phenotype scatter plot (G1) and mean of trait by groups. The graph on the left-hand side shows the dependence of phenotype values (G1) on PRS values. The graph on the right-hand side shows data stratified into 30 groups according to the PRS percentile. The Y-axis shows the average value of the trait for the group, the X-axis shows the group number.

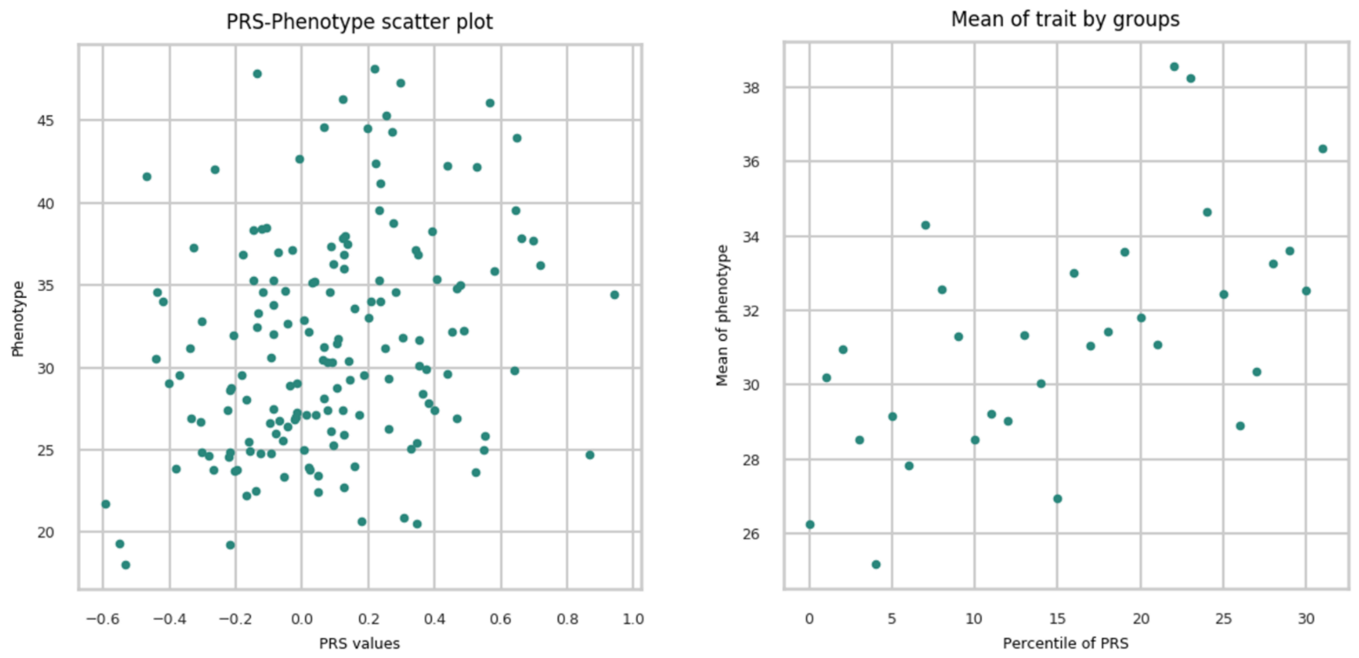

Supplementary Figure 7. PRS-Phenotype scatter plot (G2) and mean of trait by groups. The graph on the left-hand side shows the dependence of phenotype values (G2) on PRS values. The graph on the right-hand sideshows data stratified into 30 groups according to the PRS percentile. The Y-axis shows the average value of the trait for the group, the X-axis shows the group number.
