# Supplementary material for: Mapping of the gene network that regulates glycan clock of ageing
Source: Aging (Albany NY). 2023 Dec 26;15(24):14509–52. doi: 10.18632/aging.205106 (PMC10781487; doi:10.18632/aging.205106)
Supplement: Supplementary Table 5 [file aging-15-205106-s007.pdf]

Supplementary Table 5. Overview of traits and diseases in colocalization analysis.

Trait	First author	Accession number GWAS catalog	N total	Cases	Control	Case prop	Download link
Adult-onset asthma	Ferreira MAR et al	GCST007799	327253	26582	300671	0.081	ftp://ftp.ebi.ac.uk/pub/databases/gwas/summary_statistics/FerreiraMAR_30929738_GCST007799
Primary billiary cirrhosis	Cordell HJ	GCST003129	13239	2764	10475	0.209	ftp://ftp.ebi.ac.uk/pub/databases/gwas/summary_statistics/CordellHJ_26394269_GCST003129/harmonised/26394269-GCST003129-EFO_1001486-Build37.f.tsv.gz
Asthma	Han Y	GCST010042	303859	64538	239321	0.212	ftp://ftp.ebi.ac.uk/pub/databases/gwas/summary_statistics/HanY_32296059_GCST010042
Systemic Lupus Erythematosus	Julia A	GCST005831	16966	4943	8483	0.291	ftp://ftp.ebi.ac.uk/pub/databases/gwas/summary_statistics/JuliaA_29848360_GCST005831
Type I diabetes	Forgetta V	GCST010681	24840	9266	15574	0.373	ftp://ftp.ebi.ac.uk/pub/databases/gwas/summary_statistics/ForgettaV_32005708_GCST010681
IgG level	Scepanovic P	GCST006357	1000	NA	NA	NA	ftp://ftp.ebi.ac.uk/pub/databases/gwas/summary_statistics/ScepanovicP_30053915_GCST006357
Schizophrenia	Pardinas AF	GCST006803	105318	40675	64643	0.386	https://walters.psycm.cf.ac.uk/clozuk_pgc2.meta.sumstats.txt.gz
Rheumatoid arthritis	Eyre S	GCST005569	47580	13838	33742	0.290	ftp://ftp.ebi.ac.uk/pub/databases/gwas/summary_statistics/EyreS_23143596_GCST005569
Type II diabetes	Mahajan A	GCST007518	298957	48286	250671	0.162	ftp://ftp.ebi.ac.uk/pub/databases/gwas/summary_statistics/MahajanA_29632382_GCST007518/T2D_European.BMIadjusted.txt
Allergic Disease	Ferreira	GCST005038	360838	180129	180709	0.499	ftp://ftp.ebi.ac.uk/pub/databases/gwas/summary_statistics/FerreiraMA_29083406_GCST005038
Alzheimer's disease	Kunkle	NA	63926	21982	41944	0.344	https://www.niagads.org/datasets/ng00075
Lymphocyte percentage of white blood cells	Astle WJ	GCST004632	171748	NA	NA	NA	ftp://ftp.ebi.ac.uk/pub/databases/gwas/summary_statistics/AstleWJ_27863252_GCST004632
Total Cholesterol	Willer CJ	GCST002221	94595	NA	NA	NA	http://ftp.ebi.ac.uk/pub/databases/gwas/summary_statistics/GCST002001-GCST003000/GCST002221/harmonised/24097068-GCST002221-EFO_0004574-build37.f.tsv.gz
Crohn's disease	Liu JZ	GCST003044	20883	5956	14927	0.285	http://www.ibdgenetics.org/downloads.html
Ulcerative colitis	Liu JZ	GCST003045	27432	6968	20464	0.254	http://www.ibdgenetics.org/downloads.html
Inflammatory bowel disease	Liu JZ	GCST003043	34652	12882	21770	0.372	http://www.ibdgenetics.org/downloads.html
HDL cholesterol	Willer CJ	GCST002223	94595	NA	NA	NA	http://ftp.ebi.ac.uk/pub/databases/gwas/summary_statistics/GCST002001-GCST003000/GCST002223/harmonised/24097068-GCST002223-EFO_0004612-build37.f.tsv.gz
LDL	Willer CJ	GCST002222	94595	NA	NA	NA	http://ftp.ebi.ac.uk/pub/databases/gwas/summary_statistics/GCST002001-GCST003000/GCST002222/harmonised/24097068-GCST002222-EFO_0004611-build37.f.tsv.gz
Juvenile idiopathic arthritis	Hinks A	GCST005528	15872	2816	13056	0.177	http://ftp.ebi.ac.uk/pub/databases/gwas/summary_statistics/GCST005001-GCST006000/GCST005528/harmonised/23603761-GCST005528-EFO_1001999-Build37.f.tsv.gz
Osteoarthritis	Tachmazidou I	GCST007092	417596	39427	378169	0.094	http://ftp.ebi.ac.uk/pub/databases/gwas/summary_statistics/GCST007001-GCST008000/GCST007092/harmonised/30664745-GCST007092-EFO_0002506-build37.f.tsv.gz
Chronic kidney disease	Wuttke M	GCST008065	625219	64164	561055	0.103	http://ftp.ebi.ac.uk/pub/databases/gwas/summary_statistics/GCST008001-GCST009000/GCST008065/CKD_overall_EA_JW_20180223_nstud23.dbgap.txt.gz

Hypertension	Zhu Z	GCST007610	458554	144793	313761	0.316	http://ftp.ebi.ac.uk/pub/databases/gwas/summary_statistics/GCST007001-GCST008000/GCST007610/ZhuZ_30940143_ukbb.bolt_460K_selfRepWhite.doctor_highbloodpressure.assoc.gz
Thyroid cancer	Zhou W	GCST008371	407757	358	407399	0.001	http://ftp.ebi.ac.uk/pub/databases/gwas/summary_statistics/GCST008001-GCST009000/GCST008371/PheCode_193_SAIGE_MACge20.txt.vcf.gz
Lung cancer	Rashkin SR	GCST90011812	412835	2485	410350	0.006	http://ftp.ebi.ac.uk/pub/databases/gwas/summary_statistics/GCST90011001-GCST90012000/GCST90011812/harmonised/32887889-GCST90011812-EFO_0001071-Build37.f.tsv.gz
Ovarian cancer	Rashkin SR	GCST90011821	411609	1259	410350	0.003	http://ftp.ebi.ac.uk/pub/databases/gwas/summary_statistics/GCST90011001-GCST90012000/GCST90011821/harmonised/32887889-GCST90011821-EFO_0001075-Build37.f.tsv.gz
Colorectal cancer	Zhou W	GCST008372	387318	4562	382756	0.012	http://ftp.ebi.ac.uk/pub/databases/gwas/summary_statistics/GCST008001-GCST009000/GCST008372/PheCode_153_SAIGE_MACge20.txt.vcf.gz
Breast cancer	Rashkin SR	GCST90011804	428231	17881	410350	0.042	http://ftp.ebi.ac.uk/pub/databases/gwas/summary_statistics/GCST90011001-GCST90012000/GCST90011804/harmonised/32887889-GCST90011804-EFO_0000305-Build37.f.tsv.gz
COVID-19 (Hospitalized vs. Population)	COVID-19 Host Genetics Initiative	NA	1887658	9986	1877672	0.005	https://storage.googleapis.com/covid19-hg-public/20201215/results/20210107/COVID19_HGI_B2_ALL_eur_leave_23andme_20210107.b37.txt.gz

Trait- tested trait or disease; First author- first author of the publication describing the GWAS of the trait or disease; Accession No- accession number in GWAS Catalog; N total- total number of subjects in the study; Cases- number of case subjects in the study; Controls- number of control subjects in the study; Case prop- proportion of the cases in the total sample; Download link- link for download of the GWAS summary statistics for the given trait or disease.
