# Supplementary material for: Mapping of the gene network that regulates glycan clock of ageing
Source: Aging (Albany NY). 2023 Dec 26;15(24):14509–52. doi: 10.18632/aging.205106 (PMC10781487; doi:10.18632/aging.205106)
Supplement: Appendix Table 1 [file aging-15-205106-s009.pdf]

APPENDIX TABLES

Appendix Table 1. Pearson's correlation coefficients for UPLC and LC-MS derived glycan traits after applying different normalization types and weighting of the subclass-specific values in LC-MS measurements.

**UPLC**	**Total area**	**Largest peak**	**Median quotient**	**Total area**	**Total area**	**Total area**	**Total area**	**Total area**	**Total area**	**Total area**
LCMS	Total area	Largest peak	Median quotient	Total area with trait subclass average	Total area with weighted subclass average	Total area per subclass with weighted average	Total area per subclass	Total area per subclass with trait average	Total area with subclass concentration applied to raw values	Total area with response factor applied to raw values
G0	0.96	0.96	0.96	0.95	0.97	0.97	0.95	0.95	0.96	0.95
G1	0.78	0.77	0.78	0.75	0.80	0.79	0.75	0.75	0.79	0.73
G2	0.91	0.91	0.91	0.91	0.91	0.91	0.90	0.90	0.91	0.90

Combination of normalization types as applied in UPLC- and LC- MS-derived data is shown on the top.
