# Supplementary material for: Mapping of the gene network that regulates glycan clock of ageing
Source: Aging (Albany NY). 2023 Dec 26;15(24):14509–52. doi: 10.18632/aging.205106 (PMC10781487; doi:10.18632/aging.205106)
Supplement: Appendix Table 2 [file aging-15-205106-s010.docx]

Appendix Table 2. List of IgG galactosylation associated loci and comparison of GWAS summary statistics for significantly associated genomic regions in current GWAS with previous GWAS of IgG N-glycome.

| Discovery GWAS | | | | | | | | | | | | Replication GWAS | | | Previous GWAS | | | | | | |
| --- | --- | --- | --- | --- | --- | --- | --- | --- | --- | --- | --- | --- | --- | --- | --- | --- | --- | --- | --- | --- | --- |
| Locus | top SNP | top SNP chr | top SNP pos | Trait | EA | OA | EAF | Beta | StdErr | P-value | Beta repl | | SE repl | P repl | | Study | SNP | chr:pos | Trait | Pval | LD r2 with top SNP |
| 1:24699711-25493756 | rs188468174 | 1 | 25291697 | g0 | T | C | 0.015 | 0.693 | 0.048 | 3.20E-47 | 0.865 | | 0.076 | 2.96E-30 | | Klaric et al. | rs10903118 | 1:25294878 | IGP74 | 5.14E-13 | 0.01 |
| 2:26109539-26149988 | rs111919630 | 2 | 26139430 | g0 | T | C | 0.334 | -0.062 | 0.010 | 3.80E-10 | -0.049 | | 0.015 | 1.03E-03 | | Shadrina et al. | rs11895615 | 2:26113120 | Bisecting_GlcNac | 5.69E-10 | 0.87 |
| 4:103390496-103567348 | rs3774964 | 4 | 103519487 | g2 | A | G | 0.37 | -0.065 | 0.010 | 1.56E-11 | -0.038 | | 0.015 | 9.20E-03 | |  |  |  |  |  |  |
| 6:31107733-31164511 | rs1265109 | 6 | 31119589 | g0 | T | G | 0.295 | -0.063 | 0.011 | 1.57E-09 | 0.013 | | 0.019 | 4.97E-01 | | Klaric et al. | rs3099844 | 6:31448976 | IGP15 | 1.12E-13 | 0.02 |
| 6:74168723-74285118 | rs3822960 | 6 | 74230859 | g2 | T | C | 0.306 | 0.061 | 0.010 | 5.84E-10 | 0.028 | | 0.015 | 6.24E-02 | |  |  |  |  |  |  |
| 6:143088071-143206826 | rs7758383 | 6 | 143169723 | g2 | A | G | 0.486 | 0.092 | 0.009 | 6.09E-23 | 0.145 | | 0.014 | 9.44E-25 | | Klaric et al. | rs7758383 | 6:143169723 | IGP13 | 9.61E-14 |  |
| 7:150856165-150906453 | rs113745074 | 7 | 150942349 | g0 | T | C | 0.094 | 0.120 | 0.015 | 6.19E-16 | 0.134 | | 0.022 | 8.47E-10 | | Klaric et al. | rs7812088 | 7:150919829 | IGP2 | 2.06E-22 | 0.95 |
| 8:103542538-103550211 | rs13250010 | 8 | 103545983 | g0 | T | G | 0.356 | 0.063 | 0.010 | 7.71E-11 | 0.062 | | 0.015 | 3.47E-05 | | Klaric et al. | rs10096810 | 8:103545436 | IGP77 | 9.52E-11 | 0.9 |
| 9:32933492-33385427 | rs13297246 | 9 | 33128617 | g2 | A | G | 0.184 | 0.195 | 0.013 | 4.21E-55 | 0.202 | | 0.019 | 2.36E-26 | | Klaric et al. | rs10813951 | 9:33128021 | IGP17 | 8.84E-34 | 0.07 |
| 11:65555524-65555524 | rs10896045 | 11 | 65555524 | g1 | A | G | 0.301 | 0.079 | 0.013 | 2.61E-09 | 0.079 | | 0.017 | 2.42E-06 | | Shadrina et al. | rs479844 | 11:65551957 | N-glycosylation | 1.97E-13 | 0.54 |
| 17:16813994-16875636 | rs34562254 | 17 | 16842991 | g1 | A | G | 0.106 | 0.166 | 0.019 | 1.48E-18 | 0.092 | | 0.024 | 9.95E-05 | | Shadrina et al. | rs4561508 | 17:16848750 | N-glycosylation | 1.38E-10 | 0.88 |
| 17:43856639-44863413 | rs199516 | 17 | 44856485 | g0 | T | C | 0.221 | 0.068 | 0.012 | 1.20E-08 | 0.058 | | 0.018 | 1.25E-03 | | Klaric et al. | rs199456 | 17:44797919 | IGP14 | 6.76E-14 | 0.94 |
| 17:45766846-45870129 | rs1808192 | 17 | 45794706 | g2 | A | G | 0.351 | 0.061 | 0.010 | 5.94E-10 | 0.051 | | 0.015 | 6.78E-04 | | Klaric et al. | rs11651000 | 17:45835278 | IGP59 | 2.66E-12 | 0.23 |
| 17:56404349-56418136 | rs2526377 | 17 | 56410041 | g2 | A | G | 0.463 | 0.062 | 0.009 | 5.59E-11 | 0.057 | | 0.015 | 8.05E-05 | |  |  |  |  |  |  |
| 17:79158040-79268562 | rs2659005 | 17 | 79218714 | g2 | T | C | 0.436 | 0.079 | 0.009 | 4.36E-17 | 0.097 | | 0.016 | 6.99E-10 | | Klaric et al. | rs2725391 | 17:79192430 | IGP24 | 9.91E-16 | 0.74 |
| 21:36564553-36665202 | rs4817708 | 21 | 36567726 | g0 | T | C | 0.229 | 0.073 | 0.011 | 2.39E-11 | 0.043 | | 0.017 | 1.15E-02 | | Klaric et al. | rs7281587 | 21:36565278 | IGP45 | 1.13E-13 | 0.9 |

Locus- chromosome: start-end position in GRCh37 (hg19) build; top SNP- rsID identifier for the top SNP in the locus; pos- chromosomal position of the top SNP; Trait- galactosylation trait with the strongest association with the locus;EA- effect allele; OA- non-effect allele; EAF- effect allele frequency; Beta- effect estimate for the top SNP; StdErr- standard error of the effect estimate; P-value- p value of the association; Study- Latest IgG N-glycome GWA study where the association was identified; SNP- rsID identified of the top SNP in the latest IgG N-glycome GWAS where the association was identified; chr:pos- chromosomal coordinates for the SNP in GRCh37 (hg19) build; Trait- trait with the strongest association with the SNP in the genomic region in GWAS denoted in Study column; Pval- p-value of the association; LD r2 with top SNP- LD r2 value between top SNP in discovery GWAS and top SNP in previous GWAS.
