# Supplementary material for: Mapping of the gene network that regulates glycan clock of ageing
Source: Aging (Albany NY). 2023 Dec 26;15(24):14509–52. doi: 10.18632/aging.205106 (PMC10781487; doi:10.18632/aging.205106)
Supplement: Appendix Tables 3-14 [file aging-15-205106-s011.pdf]

Appendix Table 3. List of genes mapped by positional, eQTL and chromatin interaction mapping in FUMA.

Mapping strategy	Mapped genes
**Positional mapping**	*GRHL3,STPG1,NIPAL3,RCAN3,SRRM1,RUNX3,ASXL2,KIF3C,NFKB1,MANBA,MB21D1,MTO1,EEF1A1,HIVEP2,GBX1,ABCF2,CHPF2,SMARCD3,KB1980E6.3,APTX,DNAJA1,SMU1,B4GALT1,SPINK4,BAG1,CHMP5,NFX1,AQP7,AP5B1,OVOL1,TNFRSF13B,CRHR1,SPPL2C,MAPT,STH,KANSL1,ARL17B,LRRC37A,NSF,WNT3,KPNB1,TBKBP1,TBX21,BZRAP1,SUPT4H1,AZI1,ENTHD2,C17orf89,SLC38A10,RUNX1*
**eQTL mapping**	*MANBA,EEF1A1,NSF,MTO1,OOEP,DNAJA1,RAD51C,TMEM176A,TBKBP1,SUPT4H1,GPS1,CISD2,APTX,LRRC37A,WNT3,B4GALT1,LRRC37A2,C17orf89,KB-1980E6.3,SMU1,OVOL1,KANSL1,ARHGAP27,ARL17A,ARL17B*
**Chromatin interaction mapping**	*CNR2,PNRC2,SRSF10,MYOM3,IL22RA1,IFNLR1,GRHL3,STPG1,NIPAL3,RCAN3,SRRM1,SYF2,DAPP1,DNAJB14,H2AFZ,SLC39A8,NFKB1,UBE2D3,CISD2,SLC9B1,SLC9B2,BDH2,CENPE,EEF1A1,SLC17A5,FILIP1,HIVEP2,AIG1,ADAT2,AL031320.1,PEX3,FUCA2,PLAGL1,RRM2B,UBR5,ODF1,ACO1,NDUFB6,GVQW1,APTX,DNAJA1,SMU1,B4GALT1,SPINK4,NFX1,AQP3,NOL6,RNASEH2C,ZNF287,MPRIP,PLD6,RP1145M22.4,FLCN,STH,KANSL1,LRRC37A,RPRML,KPNB1,TBKBP1,TBX21,VEZF1,MKS1,LPO,MPO,RNF43,HSF5,MTMR4,RCAN1*

Appendix Table 4. Results of genome-wide gene-based association test by MAGMA (FDR<0.05).

Trait	Gene	Chr	Start	End	N SNPs	Z	P	P adjusted
g0	RUNX3	1	25226002	25291612	113	4.207	1.29E-05	9.68E-03
g2	RUNX3	1	25226002	25291612	113	4.109	1.99E-05	1.33E-02
g0	NFKB1	4	103422486	103538459	276	6.163	3.57E-10	1.33E-06
g0	MANBA	4	103552660	103682151	235	4.202	1.33E-05	9.77E-03
g2	NFKB1	4	103422486	103538459	276	6.506	3.86E-11	2.70E-07
g2	MANBA	4	103552660	103682151	235	4.749	1.02E-06	1.24E-03
g0	EEF1A1	6	74225473	74233520	19	4.370	6.21E-06	5.61E-03
g0	HIVEP2	6	143072604	143266338	486	4.459	4.11E-06	4.05E-03
g1	HIVEP2	6	143072604	143266338	485	7.134	4.86E-13	9.07E-09
g2	MTO1	6	74171301	74218959	118	4.824	7.05E-07	9.39E-04
g2	EEF1A1	6	74225473	74233520	19	5.809	3.14E-09	8.37E-06
g2	HIVEP2	6	143072604	143266338	486	6.109	5.00E-10	1.65E-06
g0	ABCF2	7	150904923	150924316	70	5.770	3.97E-09	1.01E-05
g0	CHPF2	7	150929575	150935908	16	4.928	4.16E-07	6.30E-04
g0	SMARCD3	7	150935850	150974982	113	6.517	3.58E-11	2.70E-07
g2	ABCF2	7	150904923	150924316	70	4.621	1.91E-06	2.14E-03
g2	CHPF2	7	150929575	150935908	16	3.890	5.02E-05	2.84E-02
g2	SMARCD3	7	150935850	150974982	113	5.292	6.06E-08	1.13E-04
g0	KB-1980E6.3	8	103540968	103550896	60	6.235	2.25E-10	9.55E-07
g2	KB-1980E6.3	8	103540968	103550896	60	5.039	2.34E-07	3.86E-04
g0	DNAJA1	9	33025209	33039905	55	3.791	7.49E-05	3.99E-02
g0	SMU1	9	33041762	33076665	103	4.738	1.08E-06	1.28E-03
g0	B4GALT1	9	33104080	33167354	238	6.800	5.22E-12	4.87E-08
g0	SPINK4	9	33218363	33248565	103	5.628	9.11E-09	2.22E-05
g0	BAG1	9	33247818	33264761	22	4.264	1.01E-05	7.94E-03
g0	CHMP5	9	33264940	33281977	21	5.408	3.20E-08	6.39E-05
g0	NFX1	9	33290509	33371155	115	6.090	5.64E-10	1.76E-06
g1	B4GALT1	9	33104080	33167354	239	7.576	1.79E-14	5.00E-10
g2	DNAJA1	9	33025209	33039905	55	4.353	6.72E-06	5.85E-03
g2	SMU1	9	33041762	33076665	103	5.461	2.37E-08	4.91E-05
g2	B4GALT1	9	33104080	33167354	239	6.109	5.00E-10	1.65E-06
g2	SPINK4	9	33218363	33248565	103	6.352	1.06E-10	4.95E-07
g2	BAG1	9	33247818	33264761	22	4.350	6.79E-06	5.85E-03
g2	CHMP5	9	33264940	33281977	21	5.902	1.79E-09	5.01E-06
g2	NFX1	9	33290509	33371155	115	7.088	6.80E-13	9.52E-09
g1	OVOL1	11	65554493	65564690	25	4.144	1.71E-05	1.20E-02
g0	ARHGAP27	17	43471275	43511787	103	3.784	7.73E-05	4.08E-02
g0	PLEKHM1	17	43513266	43568115	97	3.762	8.44E-05	4.42E-02
g0	CRHR1	17	43699267	43913194	957	4.787	8.47E-07	1.09E-03
g0	SPPL2C	17	43922256	43924438	18	4.712	1.23E-06	1.43E-03
g0	MAPT	17	43971748	44105700	682	4.878	5.37E-07	7.70E-04
g0	STH	17	44076616	44077060	1	4.884	5.20E-07	7.66E-04
g0	KANSL1	17	44107282	44302733	743	4.979	3.20E-07	5.11E-04
g0	ARL17B	17	44352150	44439130	14	4.583	2.30E-06	2.43E-03
g0	NSF	17	44668035	44834830	67	4.967	3.40E-07	5.29E-04
g0	WNT3	17	44839872	44910520	112	5.383	3.67E-08	7.09E-05
g0	TBX21	17	45810610	45823485	38	4.367	6.31E-06	5.61E-03
g0	AZI1	17	79163393	79196799	130	5.152	1.29E-07	2.33E-04

g0	ENTHD2	17	79202077	79212891	30	6.226	2.39E-10	9.55E-07
g0	C17orf89	17	79213039	79215081	6	5.998	9.98E-10	2.94E-06
g0	SLC38A10	17	79218800	79269347	185	5.490	2.01E-08	4.33E-05
g1	TNFRSF13B	17	16832849	16875432	180	6.406	7.49E-11	4.20E-07
g2	CRHR1	17	43699267	43913194	957	4.294	8.78E-06	7.23E-03
g2	SPPL2C	17	43922256	43924438	18	4.207	1.30E-05	9.68E-03
g2	MAPT	17	43971748	44105700	682	4.394	5.57E-06	5.20E-03
g2	STH	17	44076616	44077060	1	4.424	4.85E-06	4.61E-03
g2	KANSL1	17	44107282	44302733	743	4.515	3.16E-06	3.28E-03
g2	ARL17B	17	44352150	44439130	14	4.182	1.44E-05	1.05E-02
g2	NSF	17	44668035	44834830	67	4.608	2.03E-06	2.23E-03
g2	WNT3	17	44839872	44910520	112	5.056	2.14E-07	3.62E-04
g2	ITGB3	17	45331212	45421658	287	3.940	4.08E-05	2.38E-02
g2	ITGB3	17	45331263	45421658	287	3.940	4.08E-05	2.38E-02
g2	TBX21	17	45810610	45823485	38	4.771	9.15E-07	1.14E-03
g2	BZRAP1	17	56378592	56406152	82	4.075	2.30E-05	1.47E-02
g2	SUPT4H1	17	56422539	56430454	22	4.088	2.18E-05	1.42E-02
g2	AZI1	17	79163393	79196799	130	6.462	5.18E-11	3.22E-07
g2	ENTHD2	17	79202077	79212891	30	7.584	1.68E-14	5.00E-10
g2	C17orf89	17	79213039	79215081	6	6.366	9.68E-11	4.93E-07
g2	SLC38A10	17	79218800	79269347	185	7.012	1.18E-12	1.32E-08
g0	RUNX1	21	36160098	37376965	3508	4.784	8.60E-07	1.09E-03
g2	RUNX1	21	36160098	37376965	3506	4.440	4.51E-06	4.35E-03

Trait- galactosylation trait which was tested; Gene- symbol of the gene; Chr- chromosome on which gene is found; Start- starting position for the gene sequence in GRCh37(hg19) build; End- end position for the gene sequence; N SNPs- number of SNPs in the gene; Z- Z-score of the association; P- unadjusted P value of the association; P adjusted- adjusted p value using Benjamini and Hochberg method.

Appendix Table 5. Results of implementation of SBayesR and C+T models for G0, G1 and G2.

PRS model	Parameters	G0			G1			G2		
		R2	R spearman	PRS p-value	R2	R spearman	PRS p-value	R2	R spearman	PRS p-value
SbayesR	PRS+sex+age	0.3345	0.5861	4.68E-04	0.0159	0.1424	0.4978	0.3407	0.5776	1.76E-04
	PRS+age	0.3108	0.5796	2.70E-04	0.0056	0.1240	0.4347	0.3232	0.5745	1.29E-04
	PRS+sex	0.1076	0.2977	8.55E-04	0.0146	0.1063	0.5009	0.1008	0.2934	6.55E-04
	PRS	0.0736	0.2528	4.79E-04	0.0038	0.0574	0.4367	0.0737	0.2801	4.77E-04
C+T	PRS+sex+age	0.3372	0.6198	3.31E-04	0.0363	0.2043	0.0524	0.3423	0.5843	1.45E-04
	PRS+age	0.3020	0.6086	7.86E-04	0.0245	0.1817	0.0559	0.3191	0.5784	2.16E-04
	PRS+sex	0.0935	0.2892	3.29E-03	0.0346	0.1756	0.0543	0.0946	0.2835	1.18E-03
	PRS	0.0450	0.1999	6.74E-03	0.0222	0.1200	0.0586	0.0600	0.2217	1.67E-03
Models	age+sex	0.2807	0.5379		0.0130	0.1276		0.2791	0.5199	
	age	0.2506	0.5346		0.0018	0.1037		0.2577	0.5202	
	sex	0.0428	0.1702		0.0118	0.1026		0.0324	0.1512	

PRS Model - method that was used to build model; Parameters - sets of parameters for a linear regression model with the trait as the outcome; R2 - part of trait variance that a model explains; R Spearman - the Spearman correlation coefficient between observed and predicted values of the trait; PRS p-value - the two-tailed p-value for the t-stats of PRS param.

Appendix Table 6. Meta-analysis results of testing differences in G0, G1 and G2 traits in treated (upregulation) and control cell line.

Experiment	Glycan trait	Estimate	SE	p-value	CI lower	CI upper
VPR_EEF1A1	GP_G0	2.484280	0.966877	0.010188	0.589236	4.379323
	GP_G1	-2.246458	0.487330	0.000004	-3.201608	-1.291309
	GP_G2	-0.257913	0.352768	0.464710	-0.949325	0.433499
VPR_HIVEP2	GP_G0	-1.408927	0.518379	0.006569	-2.424931	-0.392924
	GP_G1	0.117828	0.443605	0.790535	-0.751621	0.987278
	GP_G2	1.079269	0.379913	0.004500	0.334654	1.823884
VPR_MANBA	GP_G0	2.106936	1.137043	0.063883	-0.121627	4.335500
	GP_G1	-2.521960	0.755039	0.000837	-4.001809	-1.042112
	GP_G2	-0.176833	0.265544	0.505459	-0.972904	0.343625
VPR_NFKB1	GP_G0	0.823330	1.339071	0.538653	-1.801201	3.447862
	GP_G1	-0.886318	1.267960	0.484545	-3.371473	1.598837
	GP_G2	0.022321	0.755465	0.976429	-1.458363	1.503006
VPR_TNFRSF13B	GP_G0	2.384531	0.622566	0.000128	1.164323	3.604739
	GP_G1	-1.515340	0.902826	0.093261	-3.284846	0.254166
	GP_G2	-0.706994	0.360817	0.050063	-1.414181	0.000194

Trait values were transformed to obtain normal distribution using Box-Cox method and analyzed using Student's t test. Estimate- effect size estimates; SE- standard error of estimate; p-value- p value of the meta-analysis; CI-confidence interval.

Appendix Table 7. Overview of platforms used for quantification of IgG N-glycans and sample demographics.

Cohort	Platform for glycan analysis	Reference	N total available (glycans)	N female	N male	median age (min-max)	N in GWAS
TwinsUK	UPLC	Menni et al.	4624	4282	342	54	4477
CROATIA-Korcula	UPLC and LC-MS	Pučić et al.	2478	1148	1330	57	2436
CROATIA-Split	LC-MS	Not published	973	383	590	52	920
CROATIA-Vis	LC-MS	Pučić et al.	683	394	289	57	675
VIKING	UPLC	Landini et al.	1086	645	441	51	1071
ORCADES	UPLC	Krištić et al.	1786	1082	704	54	1720
LLS	LC-MS	Ruhaak et al.	1841	974	867	59	1190
KORA F4	LC-MS	Wahl et al.	1823	935	888	62	1167
EGCUT	UPLC	Trbojević-Akmačić et al.	1108	516	592	69	483
GCKD	UPLC	Not published	4933	1965	2968	63	4933
EPIC	UPLC	Not published	3600	61.20%	38.80%	50	2406

Cohort- name of the study; Platform- platform used for quantification of IgG N-glycans; Reference- published study in which the IgG N-glycome analysis for the cohort was described; N total (glycans)- number of subjects in the cohort with measured glycans; N female- number of female subjects; N male- number of male subjects; median age- median age of participants in the cohort; N in GWAS- number of pariticipants in GWAS.

Appendix Table 8. Overview of genotyping arrays and imputation.

Cohort	Genotyping platform(s)	ID call rate	SNP call rate	HWE p	MAF	Imputation tool
TwinsUK	Illumina HumanHap300; Illumina HumanHap610Q	>95%	> 97% (MAF ≥ 5%); > 99% (1% ≤ MAF < 5%)	>1e-6	≥1%	MACH (Michigan Imputation Server v1.0.2)
EPIC	Human660W-Quad_v1_A	>97% >99%	>95%	>1e-3		Eagle2/minimac 3
	HumanCoreExome-12v1-0_B					Eagle2/minimac 3
	Illumina InfiniumOmniExpressExome-8v1-3_A DNA Analysis BeadChip				zCall threshold=7	Eagle2/minimac 3
LLS	Illumina660 W; Illumina OmniExpress	>95%	>95%	>1e-4	≥1%	IMPUTE2
CROATIA-Korcula 1,2,3	Illumina HumanHap s370CNV DUO/QUAD Phase 1(1); Illumina HumanOmniExpress Exome (2 and 3)	>97%	>98%	>1e-6	≥1%	SHAPEIT2/Sanger
CROATIA-Split	Illumina HumanHap 370CNV QUAD Phase I; Illumina HumanOmniExpress Exome	≥97%	>98%	>1e-6	≥1%	SHAPEIT2/Sanger
CROATIA-Vis	Illumina HumanHap300v1 BeadChip	>97%	>98%	>1e-6	≥1%	SHAPEIT2/Sanger
VIKING	Illumina HumanOmniExpress Exome	>97%	>98%	>1e-6	≥1% omni markers; ≥0.01% exome markers	SHAPEIT2/Sanger
EGCUT	Illumina GSAv1.0, GSAv2.0, GSAv2.0_EST	>95%	>95%	>1e-4	≥1%	Beagle v.28Sep18.793
KORA F4	Affymetrix Axiom	>97%	>98%	>5e-6	≥1%	SHAPEIT/IMPUTE
ORCADES	HumanHap300v2 Phase 1	>97%	>98%	>1e-6	≥1%	SHAPEIT2/Sanger
GCKD	Illumina Omni2.5Exome BeadChip	>97%	>96%	>1e-5	>1%	Eagle/minimac3

Cohort- name of the participating study; Genotyping platform- SNP array used for genotyping in the given cohort; ID call rate- ID call rate; SNP call rate- SNP call rate; HWE p- p value of HWE test used for filtering; MAF- minor allele frequency threshold used for filtering; Imputation tool- software used for imputation of genetic data.

Appendix Table 9. Glycan names and description of the most abundant glycan structure in each peak as measured by ultra-performance liquid chromatography; glycan name- name of the glycan; description- structure of the glycan (F- core fucosylated, A1- monoantennary, A2- diantennary, B- bisecting GlcNAc, G1- monogalactosylated, G2- digalactosylated, S1- monosialylated, S2- disialylated, M5- pentamannose, [3] or [6]- position of the attached galactose).

	Glycan name	Description
UPLC measured glycan traits	GP1	FA1 glycan
	GP2	A2 glycan
	GP3	Structure not determined
	GP4	FA2 glycan
	GP5	M5 glycan
	GP6	FA2B glycan
	GP7	A2G1 glycan
	GP8	FA2[6]G1 glycan
	GP9	FA2[3]G1 glycan
	GP10	FA2[6]BG1 glycan
	GP11	FA2[3]BG1 glycan
	GP12	A2G2 glycan
	GP13	A2BG2 glycan
	GP14	FA2G2 glycan
	GP15	FA2BG2 glycan
	GP16	FA2G1S1 glycan
	GP17	A2G2S1 glycan
	GP18	FA2G2S1 glycan
	GP19	FA2BG2S1 glycan
	GP20	Structure not determined
	GP21	A2G2S2 glycan
	GP22	A2BG2S2 glycan
	GP23	FA2G2S2 glycan
	GP24	FA2BG2S2 glycan

Appendix Table 10. Glycan names and description of glycan structure in each peak as measured by liquid chromatography coupled with mass spectrometry; glycan name- name of the glycan in LC-MS data; description- structure of the glycan (F- core fucosylated, N- bisecting GlcNAc, G1- monogalactosylated, G2- digalactosylated, S1- monosialylated).

Glycan class	Glycan name	Description
Total IgG1 glycans	LC_IGP1	IgG1_G0
	LC_IGP2	IgG1_G0F
	LC_IGP3	IgG1_G0FN
	LC_IGP4	IgG1_G0N
	LC_IGP5	IgG1_G1
	LC_IGP6	IgG1_G1F
	LC_IGP7	IgG1_G1FN
	LC_IGP8	IgG1_G1FNS1
	LC_IGP9	IgG1_G1FS1
	LC_IGP10	IgG1_G1N
	LC_IGP11	IgG1_G1NS1
	LC_IGP12	IgG1_G1S1
	LC_IGP13	IgG1_G2
	LC_IGP14	IgG1_G2F
	LC_IGP15	IgG1_G2FN
	LC_IGP16	IgG1_G2FNS1
	LC_IGP17	IgG1_G2FS1
	LC_IGP18	IgG1_G2N
	LC_IGP19	IgG1_G2NS1
	LC_IGP20	IgG1_G2S1
Total IgG2 glycans	LC_IGP87	IgG2_G0
	LC_IGP88	IgG2_G0F
	LC_IGP89	IgG2_G0FN
	LC_IGP90	IgG2_G0N
	LC_IGP91	IgG2_G1
	LC_IGP92	IgG2_G1F
	LC_IGP93	IgG2_G1FN
	LC_IGP94	IgG2_G1FNS1
	LC_IGP95	IgG2_G1FS1
	LC_IGP96	IgG2_G1N
	LC_IGP97	IgG2_G1NS1
	LC_IGP98	IgG2_G1S1
	LC_IGP99	IgG2_G2
	LC_IGP100	IgG2_G2F
	LC_IGP101	IgG2_G2FN
	LC_IGP102	IgG2_G2FNS1
	LC_IGP103	IgG2_G2FS1
	LC_IGP104	IgG2_G2N
	LC_IGP105	IgG2_G2NS1
	LC_IGP106	IgG2_G2S

Total IgG4 glycans	LC_IGP173	IgG4_G0F
	LC_IGP174	IgG4_G0FN
	LC_IGP175	IgG4_G1F
	LC_IGP176	IgG4_G1FN
	LC_IGP177	IgG4_G1FNS1
	LC_IGP178	IgG4_G1FS1
	LC_IGP179	IgG4_G2F
	LC_IGP180	IgG4_G2FN
	LC_IGP181	IgG4_G2FNS1
	LC_IGP182	IgG4_G2FS1

Appendix Table 11. Formulas used for calculation of G0, G1 and G2 traits from LC-MS and UPLC measured glycan data.

IgG N-glycan trait	Description	LCMS formula	UPLC formula
G0	Percentage of agalactosylated structures in total IgG N-glycome	(LC_IGP1+LC_IGP2+LC_IGP3+LC_IGP4+LC_IGP87+LC_IGP88+LC_IGP89+LC_IGP90+LC_IGP173+LC_IGP174)/SUM(ALL)*100	SUM(GP1+GP2+GP4+GP5+GP6)/SUM(GP1:GP24)*100
G1	Percentage of monogalactosylated structures in total IgG N-glycome	(SUM(LC_IGP5:LC_IGP12)+SUM(LC_IGP91:LC_IGP98)+SUM(LC_IGP175:LC_IGP178))/SUM(ALL)*100	(GP7+GP8+GP9+GP10+GP11+GP16)/SUM(GP1:GP24)*100
G2	Percentage of digalactosylated structures in total IgG N-glycome	(SUM(LC_IGP13:LC_IGP20)+SUM(LC_IGP99:LC_IGP106)+SUM(LC_IGP179:LC_IGP182))/SUM(ALL)*100	(GP12+GP13+GP14+GP15+GP17+GP18+GP19+GP21+GP22+GP23+GP24)/SUM(GP1:GP24)*100

Appendix Table 12. Parameters for SBayesR model for PRS calculation.

--pi	0.95,0.02,0.02,0.01
--gamma	0.0,0.01,0.1,1
--hsq	0.5
--chain-length	3000
--burn-in	1000
--unscale-genotype	yes
--exclude-mhc	yes
--robust	yes
--p-value	0.9

Appendix Table 13. gRNA sequences for targeted upregulation/downregulation.

sgRNA molecule	Target site sequence (5'→3')	Experiment
KIF3C_A-g1	GTGTCCGAGGATGGTGGCTCG	
KIF3C_A-g2	GTTCCCGGCATGCTCGGGCTCG	
KIF3C_A-g3	GTTGTAGTCCTGCCTTTGTTAA	
NFKB1_A-g1	GTATGCTCTCTCGACGTCAGT	
NFKB1_A-g2	GTCGAGAGAGCATACAGACAG	
NFKB1_A-g3	GGGCGGGAGGTAGGGTCGCTG	
MANBA_A-g1	GACCCGCGACGCCCGGCTTTCA	
MANBA_A-g2	GAGCGCTCAGCTGACCTAGGG	
MANBA_A-g3	GTCAGTACATACAACTGGAGGC	
SLC38A10_A-g1	GGTTTGGTGGGACGCGAGCCA	
SLC38A10_A-g2	GTCCAAACCGAGGCCTCGCGGG	CRISPRa (dSaCas9)
SLC38A10_A-g3	GCCAGCTCGTTGAACTGGGGCC	
TNFRSF13B_A-g1	GTGTGTACCCCATGGGACGCC	
TNFRSF13B_A-g2	GTTAGTGCTTGGGCTGCACTTC	
TNFRSF13B_A-g3	GCTTGCGATGAGGTATTGACTA	
EEF1A1_A-g1	GCCTCAGTGATGACGAGAGCGG	
EEF1A1_A-g2	GTGAGGCTCCGGTGCCCGTCA	
EEF1A1_A-g3	GTCGGCAATTGAACCGGTGCC	
HIVEP2_A-g1	GTCATCTGCATAGATCCGCGGC	
HIVEP2_A-g2	GGAGCTTGTTTCACTTTATAT	
HIVEP2_A-g3	GCTGCTTTGCGTAATGTCCTGG	
KIF3C_G-g1	GCCAGCTAAGCCTCCCGACTG	
KIF3C_G-g2	GCGGCATGCTCGGGCTCGGTG	
KIF3C_G-g3	GCCTGTCATCCCGGAGCGGT	
NFKB1_G-g1	GGAAGAGGAGGTTTCGCCAC	
NFKB1_G-g2	GGCGGCCCGACCGACGGGAA	
NFKB1_G-g3	GCGCCGCCTTACTGGCCGGG	
MANBA_G-g1	GCTGTAACTGAGCTCCGCGG	
MANBA_G-g2	GTCGATCTCTCCACATCTCGG	
MANBA_G-g3	GCCTGATCCAGGTATGGCGTG	
SLC38A10_G-g1	GTCCCACCAAACCCTACCCCC	
SLC38A10_G-g2	GGTCACAGGTCCCAACGTCC	CRISPRi (dSpCas9)
SLC38A10_G-g3	GGTGTCCCTAGCTGGCTCTG	
TNFRSF13B_G-g1	GCTGGACCTTGCAACCCCCA	
TNFRSF13B_G-g2	GCCCAGGCCACTCATTACTC	
TNFRSF13B_G-g3	GCGAGGTGGCCGGAGCCGTG	
EEF1A1_G-g1	GGTGCCACCAGATTCGCACG	
EEF1A1_G-g2	GTTGCGAAAAAGAACGTTCA	
EEF1A1_G-g3	GAAGGGCCATAACCCGTAAAG	
HIVEP2_G-g1	GCTTTGCGTAATGTCCTGGG	
HIVEP2_G-g2	GGCCCGTTGTCAGGTAGCGG	
HIVEP2_G-g3	GCGACGTGCACCGGCGCGCA	
NT-sgRNA	GTAGGCGCGCCGCTCTCTAC	Non-targeting control

Appendix Table 14. qPCR primer sequences for SYBR detection of gene expression.

Primer name	Sequence (5'→3')
KIF3C_FW1	CATGGAGAGGCTCATGCGAT
KIF3C_REV1	TGGTTGTCACTCATGGTCCG
NFKB1_FW1	GAAGCACGAATGACAGAGGC
NFKB1_REV1	GCTTGGCGGATTAGCTCTTTT
MANBA_FW1	TGAGCTGCGTTTCCAGTCAG
MANBA_REV1	ACATGGCATTCACCCTTCTGC
SLC38A10_FW1	GGTCCCAACACAGGCTACTG
SLC38A10_REV1	GACACCAAACAGCATCCACC
TNFRSF13B_FW1	GCAAGGAGCAAGGCAAGTTC
TNFRSF13B_REV1	TCCTTGGTACCTTCCCGAGT
EEF1A1_FW1	TTGGACACGTAGATTCGGGC
EEF1A1_REV1	CGTTCACGCTCAGCTTTCAG
HIVEP2_FW1	GGGACACAGCTCTAGGACAAA
HIVEP2_REV1	TGCGGTGGATATTGCTTCTCT
